# Characteristic analysis of α-fetoprotein-producing gastric carcinoma in China

**DOI:** 10.1186/1477-7819-11-246

**Published:** 2013-10-01

**Authors:** Xiao-Dong Li, Chang-Ping Wu, Mei Ji, Jun Wu, Binfeng Lu, Hong-Bing Shi, Jing-Ting Jiang

**Affiliations:** 1 Department of Tumor Biological Treatment, The Third Affiliated Hospital of Soochow University, 185 Juqian Street, Changzhou, Jiangsu Province, People’s Republic of China; 2 Department of Oncology, The Third Affiliated Hospital of Soochow University, 185 Juqian Street, Changzhou, Jiangsu Province, People’s Republic of China; 3 Department of Immunology, University of Pittsburgh School of Medicine, 200 Lothrop Street E1047, Pittsburgh, PA 15261, USA

**Keywords:** AFP, α-Fetoprotein-producing gastric cancer (AFGPC), Clinical observation, Prognosis

## Abstract

α-Fetoprotein-producing gastric cancer (AFPGC) is a rare type of gastric cancer. The largest population of patients with AFPGC is found in China. In the present study, a total of 4,779 GC patients, including 317 AFPGC patients, from 11 clinical studies in China with a general AFPGC/GC ratio of 6.63% were summarized and analyzed. On the basis of analysis of the clinical data, the patients with AFPGC had larger tumor size, weaker cell differentiation, worse histopathological types, deeper serosal infiltration, more lymph node and liver metastases, poorer stages, shorter survival time and more positive expression of vascular endothelial growth factors than the patients without AFPGC. Our observation is consistent with previous results reported in studies of AFPGC. Overall, AFPGC is a subtype of GC with a poor prognosis.

## Reviews

α-Fetoprotein (AFP), initially identified from human fetal tissue, is normally produced in some fetal organs, proliferating hepatocytes and some adult cancer cells, including hepatocellular carcinoma cells and yolk sac tumor cells [1]. However, serum AFP levels sometimes are elevated in patients with primary gastric cancer (GC) as well [2]. The GC with a high level of AFP is termed α-fetoprotein-producing gastric cancer (AFPGC).

The first case of AFPGC was reported by Bourreille *et al.*[3]. AFPGC comprises 2.7% to 8.0% of all GCs [4]. In the past several decades, scientists have given increasing attention to AFPGC, but to date there still have been only a few clinical studies with small sample sizes and sporadic cases of AFPGC. Most of these studies and cases were reported by Japanese physicians. Most cases were characterized by a high rate of metastasis to the liver and lymph nodes. AFPGC is known to frequently cause multiple liver metastases and to carry an extremely poor prognosis [5-7]. However, there is still no standardized process for the treatment of patients with AFPGC.

China has the largest population of patients with GC, which implies that the majority of patients with AFPGC are Chinese. Thus, it is necessary and valuable to pay attention to clinical observations of AFPGC. Unfortunately, AFPGC studies are still limited, and most of these studies are case reports. There are few reports concerning the clinicopathology or prognosis of AFPGC. Additionally, there is still no published systematic review or meta-analysis.

On the basis of the considerations mentioned above, we collected the data of clinical studies of AFPGC in China with the purpose of providing an overview of the epidemiology, pathology and clinical prognosis of AFPGC.

### Data collection

We chose the VIP database of Chinese scientific and technological journals (http://en.cqvip.com/), the largest comprehensive literature database in China, to search the literature. We entered the keywords “AFPGC” or “jia tai dan bai AND wei ai” (the equivalent Chinese phrases for α-fetoprotein AND gastric cancer using Chinese characters) and searched for clinical studies in which patients with AFP-positive GC were examined. We have completed the preliminary screening and obtained 55 articles. From among these articles, we selected six randomized controlled studies and five noncontrolled retrospective studies. These studies were published in the following medical journals with the ISSNs and number of studies from each: *Chinese Journal of Current Advances in General Surgery* (ISSN 1009–9905; *n* = 1), *Journal of Modern Oncology* (ISSN 1672–4992; *n* = 1), *Zhejiang Medical Journal* (ISSN 1006–2785; *n* = 1), *Journal of Chinese Physician* (ISSN 1006–2440; *n* = 1), *Medical Journal of Communications* (ISSN 1006–2440; *n* = 2), *Jiangxi Medical Journal* (ISSN 1006–2238; *n* = 1), *Journal of Clinical & Experimental Pathology* (ISSN 2161–0681; *n* = 1), *Modern Journal of Integrated Traditional Chinese and Western Medicine* (ISSN 1008–8849; *n* = 1), *Journal of Practical Oncology* (ISSN 1001–1692; *n* = 1) and *Chinese Clinical Oncology* (ISSN 2304–3873; *n* = 1).

These studies are published in Chinese, but the abstracts of these articles are in both Chinese and English. The studies that were published in English in Chinese journals were excluded. The following 11 articles were included (studies 1 through 6 were randomized controlled studies, and studies 7 through 11 were noncontrolled retrospective studies):

 1. Tian L, Yao K, Wu A, Li L, Zhang Z, Pei, T, Liu L: **Retrospective analysis of 32 α-fetoprotein producing gastric cancer cases.***Chin J Curr Adv Gen Surg* 2011, **14:**441–453.

 2. Mi H, Zhao X, Yang Y: **Expression of AFP in gastric carcinoma and its relationship with VEGF.***Mod Oncol* 2011, **19:**106–108.

 3. Wang F, Zheng Z: **The clinicopathological features of α-fetoprotein positive gastric cancer.***Zhejiang Med J* 2004, **26:**895–896.

 4. Li B, Luo J, Li J: **Clinical significance of α-fetoprotein (AFP) expression in gastric carcinoma patients.***J Chin Physician* 2007, **9:**606–607.

 5. Shi Y, Chou A, Wang X: **Clinical significance of α-fetoprotein in gastric carcinoma patients.***Med J Commun* 2006, **20:**698–700.

 6. Peng Z, Xiong J: **The analysis of the relationship between serum AFP and the clinicopathology of the stomach.***Jiangxi Med J* 2009, **44:**301–304.

 7. Li X, Shi F, Le, M, *et al*.: **A study of histopathology and classification on AFP-positive gastric carcinoma.***J Clin Exp Pathol* 1999, **15:**293–295.

 8. Chen X, Ji Z, Lou Y: **Clinical characteristics of α-fetoprotein producing gastric cancer.***Mod J Integr Tradit Chin West Med* 2004, **13:**2164.

 9. Cao Y, Pan L: **Analysis of 62 cases of α-fetoprotein producing gastric cancer.***Med J Communic* 2003, **17:**514–515.

 10. **Zheng ZC, Wang SB, Cui XD, Xu HJ, Zhang PF: A study of the correlation of serum AFP and biological behaviors in gastric cancer.***J Pract Oncol* 1997, **11:**45–47.

 11. **Hu YQ, Chen HQ, Xu T, Liang ZC, Mao M: Clinical analysis of gastric carcinoma with high serum level of α-fetoprotein.***Chin Clin Oncol* 2007, **12:**446–448.

The following data were collected from the 11 articles: names and addresses of the authors, title, journal, volume, issue, page range, time range of patients, number of patients (males and females), median (or average) age range, site of tumor, diameter of tumor, Borrmann type, histopathology, differentiation degree, status of vascular invasion, surgery, stage of disease (TNM), inclusion and exclusion criteria, values of tumor markers and follow-up. After evaluation of the 11 studies, the information mentioned above was recorded and the results were compared. A special emphasis was placed on histopathology, differentiation degree, vascular invasion status, surgery, disease stage and outcomes of follow-up. After the evaluation was finished, a summary form encompassing the 11 studies and all information concerning indices was completed.

### Evaluation of patient characteristics

The 11 studies were conducted in various parts of China. In these 11 studies, an aggregate total of 4,779 GC patients were enrolled. From among these 4,779 GC cases, 317 were AFPGCs. The general AFPGC/GC ratio was 6.63%, which was consistent with the results from other countries and suggested the annual incidence of AFPGC was 2% to 3%. Thus, it is necessary to examine AFP in sera or tissues from patients with GC.

These 317 AFPGC cases included 200 males and 61 females. The gender of the other 56 patients was not reported in the literature. In the nine studies with data for age, the age range was 19 to 82 years old. In the 10 studies with the median or average age, the median or average age was in the range of 55 to 62.2 years old. Therefore, the gender and age are not the correlation factors of AFPGC, so that the two studies that did not report patients’ gender and the one study that did not report patients’ age were still taken into consideration in the assessment.

### Evaluation of pathological and histological conditions

Information related to tumor locations is available in six of the studies (studies 2, 5, 6, 7, 8 and 11). In these six studies, 179 AFPGC cases were included. From among these cases, the tumors from 93 cases were located in the antrum (51.9%), 22 were in the body of the stomach (12.3%), 49 were in the cardia or bottom of the stomach (27.4%) and 15 were in the whole stomach (8.4%).

Information related to the diameters of tumors is available in six studies (studies 1, 2, 3, 5, 6 and 10). Because of the different ways of reporting these data, the studies were evaluated twice. In studies 1, 2, 3, 5 and 10, there were a total of 93 AFPGC cases. Among these cases, there were 20 cases with the tumor diameters less than 3 cm (21.5%), and the other 73 cases with tumor diameters more than 3 cm (78.5%). In studies 1 and 6, there were totally 53 AFPGC cases. Among these 53 cases, there were 34 with tumor diameters less than 5 cm (64.2%), and the other 19 had tumor diameters more than 5 cm (35.8%).

Information correlated with Borrmann type was available in five studies (studies 1, 2, 3, 5 and 10). In these five studies, there were 174 AFPGC cases. Among these cases, there were 77 classified as Borrmann types I and II (44.3%) and 97 classified as Borrmann types III and IV (55.7%).

Information related to histopathological type was available in three studies (studies 1, 3 and 9). In these three studies, there was an aggregate a total of 112 AFPGC cases. From among these cases, 76 cases were adenomas (9 cases of papillary cystadenocarcinoma, 16 cases of tubular adenocarcinoma, 3 cases of mucinous adenocarcinoma and 48 unknown) (67.9%), 15 cases were signet ring cell carcinoma (13.4%), 7 were medullary carcinoma (6.2%) and 14 were undifferentiated carcinomas (12.5%).

Information correlated with differentiation degree was available in five studies (studies 1, 2, 5, 6 and 8). In these five studies, there was an aggregate total of 106 AFPGC cases. From among these cases, 19 cases had strong or moderate differentiation (17.9%) and 87 had poor or no differentiation (82.1%). Information related to vascular invasion was available in two studies (studies 1 and 7). In these two studies, there was an aggregate total of 95 AFPGC cases. From among these cases, 60 had vascular invasion (63.2%) and 35 did not (36.8%).

### Evaluation of clinical stage conditions

Information related to serosal infiltration (T) was available in five studies (studies 3 through 6 and study 10). In these five studies, there was an aggregate total of 77 AFPGC cases. From among these cases, 29 did not have serosal penetration (T1 or T2) (37.7%) and 48 did (T3 or T4) (62.3%).

Information correlated with lymph node metastasis (N) was available in eight studies (studies 1 through 8). In these eight studies, there was an aggregate total of 203 AFPGC cases. From among these cases, 27 cases had no lymph node metastasis (N0) (13.3%) and 176 did (N1 through N3) (86.7%). In three of the eight studies (studies 2 through 4), the number of metastatic lymph nodes was available. In these three studies, there was an aggregate total of 55 AFPGC cases. From among these cases, 6 did not involve lymph node metastasis (10.9%), 7 had metastasis of 1 to 4 lymph nodes (12.7%) and 42 had metastasis of more than 4 lymph nodes (76.4%).

Information associated with liver metastasis was available in nine studies (studies 1 through 9). In these 9 studies, there was an aggregate total of 265 AFPGC cases. From among these cases, 144 involved liver metastasis (54.3%) and 121 did not (45.7%).

Information correlated with the disease stages was available in six studies (studies 1 through 4 and studies 10 and 11). In these 6 studies, there was an aggregate total of 139 AFPGC cases. From among these cases, 9 cases were at stage I (6.5%), 19 were at stage II (13.7%), 33 were at stage III (23.7%) and 78 were at stage IV (56.1%).

### Evaluation of patient outcomes

Information correlated with median survival time (MST) was available in two studies (studies 1 and 5). The MSTs were 35.7 and 18.7 months, respectively, and the overall MST was 31.1 months. Information correlated with the 1-year, 3-year and 5-year survival rates was available in 4, 4 and 5 studies, respectively. The 1-year survival rate was in the range of 47.6% to 92.1% (studies 2 through 4 and study 11). The 3-year survival rate was in the range of 8.9% to 58.3% (studies 2 through 4 and study 11). The 5-year survival rate was in the range of 0% to 49.8% (studies 1 through 5). The overall 1-year, 3-year and 5-year survival rates were 53.1% (*n* = 97), 18.7% (*n* = 97) and 8.7% (*n* = 99), respectively.

### Evaluation of other parameters

Information correlated with the expression of vascular endothelial growth factor (VEGF) was available in only study 2. There was a total of 19 cases with VEGF-positive expression among these total 21 AFPGC cases (90.5%). Information correlated with AFP level, CEA level and hepatitis B virus (HBV) expression, and overall survival time was not statistically significant.

### Evaluation of gender

Information correlated with gender was available in three studies (studies 1, 2 and 6). In these three studies, there were aggregate totals of 74 AFPGC cases and 808 non-AFPGC cases. From among these cases, 54 AFPGC patients and 579 non-AFPGC patients were male (73.0% vs. 71.7%, respectively), and 20 AFPGC patients and 229 non-AFPGC patients were female (27.0% vs. 28.3%, respectively).

### Evaluation of pathological and histological conditions

Information related to the tumor sites was available in three studies (studies 2, 5 and 6). These three studies comprised aggregate totals of 54 AFPGC cases and 483 non-AFPGC cases. From among these cases, 23 were AFPGC cases and 144 were non-AFPGC cases with observed tumors in the antrum (42.5% vs. 29.8%, respectively), 15 were AFPGC cases and 156 were non-AFPGC cases with tumors observed in the body of the stomach (27.8% vs. 32.3%, respectively), 15 AFPGC cases and 177 non-AFPGC cases had tumors observed in the cardia or bottom of the stomach (27.8% vs. 36.7%, respectively) and 1 AFPGC and 6 non-AFPGC cases with tumors were observed in the whole stomach (1.9% vs. 1.2%, respectively).

Information correlated with the tumor diameters was available in five studies (studies 1 through 3 and studies 5 and 6). Because of the different ways of reporting these data, the studies were evaluated twice. In studies 1 through 3 and study 5, there were 83 AFPGC cases and 989 non-AFPGC cases. From among these cases, 19 were AFPGC cases and 373 were non-AFPGC cases with tumor diameters less than 3 cm (22.9% vs. 37.7%, respectively) and 64 AFPGC cases and 616 non-AFPGC cases with tumor diameters larger than 3 cm (77.1% vs. 62.3%, respectively). In addition, in studies 1 and 6, there were aggregate totals of 53 AFPGC cases and 567 non-AFPGC cases. From among these cases, 34 were AFPGC cases and 487 were non-AFPGC cases with tumor diameters less than 5 cm (64.2% vs. 85.9%, respectively) and 19 AFPGC cases and 80 non-AFPGC cases with tumor diameters larger than 5 cm (35.8% vs. 14.1%, respectively).

Information related to Borrmann type was available in two studies (studies 2 and 3). In these two studies, there were aggregate totals of 39 AFPGC cases and 442 non-AFPGC cases. From among these cases, there were aggregate totals of 16 AFPGC cases and 197 non-AFPGC cases belonging to Borrmann types I and II (41.0% vs. 44.6%, respectively) and 23 AFPGC cases and 245 non-AFPGC cases belonging to Borrmann types III and IV (59.0% vs. 55.4%, respectively).

Information correlated with histopathological type was available in one study (study 1). In that study, there were aggregate totals of 32 AFPGC cases and 436 non-AFPGC cases. From among these cases, there were aggregate totals of 3 AFPGC cases and 212 non-AFPGC cases with papillary cystadenocarcinoma (9.4% vs. 48.6%, respectively), 11 AFPGC cases and 45 non-AFPGC cases with tubular adenocarcinoma (34.3% vs. 10.3%, respectively), 3 cases AFPGC cases and 94 non-AFPGC cases with mucinous adenocarcinoma (9.4% vs. 21.6%, respectively), 6 AFPGC cases and 57 non-AFPGC cases with signet ring cell carcinoma (18.8% vs. 13.1%, respectively) and 9 AFPGC cases and 28 non-AFPGC cases with undifferentiated carcinoma (28.1% vs. 6.4%, respectively).

Information correlated with differentiation degree was available in four studies (studies 1, 2, 5 and 6). In these four studies, there were aggregate totals of 86 AFPGC cases and 919 non-AFPGC cases. From among these cases, there were 12 AFPGC cases and 290 non-AFPGC cases with strong or moderate differentiation (14.0% vs. 31.6%, respectively) and 74 AFPGC cases and 629 non-AFPGC cases with poor or no differentiation (86.0% vs. 68.4%, respectively).

### Evaluation of clinical stages

Information correlated with serosal infiltration (T) was available in four studies (studies 3 through 6). In these four studies, there were aggregate totals of 67 AFPGC cases and 614 non-AFPGC cases. From among these cases, there were aggregate totals of 26 AFPGC cases and 285 non-AFPGC cases without serosal penetration (T1 or T2) (38.8% vs. 46.4%, respectively) and 41 AFPGC cases and 329 non-AFPGC cases with serosal penetration (T3 or T4) (61.2% vs. 53.6%, respectively).

Information correlated with lymph node metastasis (N) was available in six studies. In those six studies, there were aggregate totals of 120 AFPGC cases and 1,291 non-AFPGC cases. From among those cases, there were 18 AFPGC cases and 492 non-AFPGC cases without lymph node metastasis (N0) (15.0% vs. 38.1%, respectively) and 102 AFPGC cases and 799 non-AFPGC cases with lymph node metastasis (N1 to N3) (85.0% vs. 61.9%, respectively). The number of metastatic lymph nodes was available in three of these six studies (studies 2 through 4). In those three studies, there were aggregate totals of 55 AFPGC cases and 613 non-AFPGC cases. From among these cases, there were aggregate totals of 6 AFPGC cases and 235 non-AFPGC cases without lymph node metastasis (10.9% vs. 38.3%, respectively), 7 AFPGC cases and 212 non-AFPGC cases with metastasis of 1 to 4 lymph nodes (12.7% vs. 34.6%, respectively) and 42 AFPGC cases and 166 non-AFPGC cases with metastasis of more than 4 lymph nodes (76.4% vs. 27.1%, respectively).

Information related to liver metastasis was available in these six studies. In these six studies, there were aggregate totals of 120 AFPGC cases and 1291 non-AFPGC cases. From among these cases, there were 68 AFPGC cases and 255 non-AFPGC with liver metastasis (56.7% vs. 19.8%, respectively) and 52 AFPGC cases and 1036 cases without liver metastasis (43.3% *vs.* 80.2%, respectively).

Information correlated with disease stages was available in four studies (studies 1 through 4). In these 4 studies, there were aggregate totals of 87 AFPGC cases and 1,049 non-AFPGC cases. From among these cases, there were 4 AFPGC cases and 198 non-AFPGC cases at stage I (4.6% vs. 18.9%, respectively), 14 AFPGC cases and 366 non-AFPGC cases at stage II (16.2% vs. 34.9%, respectively), 23 AFPGC cases and 325 non-AFPGC cases at stage III (26.4% *vs.* 31.0%) and 46 AFPGC cases and 160 non-AFPGC cases at stage IV (52.8% vs. 15.2%, respectively).

### Evaluation of patient outcome

Information correlated with MST was available in one study (study 5). In that study, there were 12 AFPGC cases and 111 non-AFPGC cases. The mean MSTs were 18.7 months for the AFPGC group and 41.3 months for the non-AFPGC group. The 1-year survival rate was reported in two studies (studies 2 and 4). The overall 1-year aggregated survival rates were 58.0% for AFPGC groups and 91.4% for the non-AFPGC groups, respectively. The 3-year survival rate was reported in two studies (studies 2 and 4). The overall aggregated 3-year survival rates were 11.3% for the AFPGC groups and 60.1% for the non-AFPGC groups, respectively. The 5-year survival rate was reported in three studies (studies 2, 4 and 5). The overall aggregated 5-year survival rates were 9.4% for the AFPGC groups and 31.3% for the non-AFPGC groups, respectively.

### Evaluation of other parameters

Information associated with VEGF expression was available in only one study (study 2). In that study, there were 19 cases with VEGF-positive expression from among the 21 AFPGC cases and 162 cases with VEGF-positive expression from among the 241 non-AFPGC cases (90.5% vs. 67.2%, respectively). Information related to vascular invasion, AFP level, CEA level, HBV expression, overall survival time and MST was not statistically significant. The clinical traits of AFPGC and non-AFPGC are shown in Table 1, and survival information is given in Table 2.

**Table 1:** **Comparison of clinical traits of α-fetoprotein-producing gastric cancer and non-α-fetoprotein-producing gastric cancer patients**^**a**^

**Characteristics**	**AFPGCs, *n *(%)**	**Non-AFPGCs, *n *(%)**	**Studies, *n *(study number)**
Gender, *N*	74	808	3 (1, 2, 6)
Male	54 (73.0)	579 (71.7)	
Female	20 (27.0)	229 (28.3)	
Tumor site	54	483	3 (2, 5, 6)
Antrum	23 (42.5)	144 (29.8)	
Body of stomach	15 (27.8)	156 (32.3)	
Cardia or bottom of stomach	15 (27.8)	177 (36.7)	
Whole stomach	1 (1.9)	6 (1.2)	
Diameter of tumor,^b^*N*	83	989	5 (1 to 3, 5, 6)
<3 cm	19 (22.9)	373 (37.7)	
>3 cm	64 (77.1)	616 (62.3)	
Diameter of tumor,^b^*N*	53	567	2 (1, 6)
<5 cm	34 (64.2)	487 (85.9)	
>5 cm	19 (35.8)	80 (14.1)	
Borrmann type, *N*	39	442	2 (2, 3)
I or II	16 (41.0)	197 (44.6)	
III or IV	23 (59.0)	245 (55.4)	
Histopathological type, *N*	32	436	1 (1)
Papillary cystadenocarcinoma	3 (9.4)	212 (48.6)	
Tubular adenocarcinoma	11 (34.3)	45 (10.3)	
Mucinous adenocarcinoma	3 (9.4)	94 (21.6)	
Signet ring cell carcinoma	6 (18.8)	57 (13.1)	
Undifferentiated	9 (28.1)	28 (6.4)	
Differentiation degree, *N*	86	919	4 (1, 2, 5, 6)
Well or moderate	12 (14.0)	290 (31.6)	
Poor or none	74 (86.0)	629 (68.4)	
Serosal infiltration *N*	67	614	4 (3 to 6)
T1 or T2	26 (38.8)	285 (46.4)	
T3 or T4	41 (61.2)	329 (53.6)	
Lymph node metastasis,^c^*N*	120	1291	6 (1 to 6)
No	18 (15.0)	492 (38.1)	
Yes	102 (85.0)	799 (61.9)	
Lymph node metastasis,^c^*N*	55	613	3 (2 to 4)
0	6 (10.9)	235 (38.3)	
1 - 4	7 (12.7)	212 (34.6)	
> 4	42 (76.4)	166 (27.1)	
Liver metastasis, *N*	120	1291	6 (1 to 6)
Yes	68 (56.7)	255 (19.8)	
No	52 (43.3)	1,036 (80.2)	
Stage, *N*	87	1049	4 (1 to 4)
I	4 (4.6)	198 (18.9)	
II	14 (16.2)	366 (34.9)	
III	23 (26.4)	325 (31.0)	
IV	46 (52.8)	160 (15.2)	
VEGF expression, *N*	21	241	1 (2)
Positive	19 (90.5)	162 (67.2)	

^a^AFPGC, α-fetoprotein-producing gastric cancer; VEGF, vascular endothelial growth factor. ^b^Because of the different ways of reporting these data, the studies were evaluated twice. In studies 1 through 3 and study 5, the cut-off diameter is 3 cm. In studies 1 and 6, the cut-off diameter is 5 cm. ^c^In studies 2 through 4, the number of metastatic lymph nodes is available. In other studies, the number is not available.

**Table 2:** Comparison of survival of α-fetoprotein-producing gastric cancer and non-α-fetoprotein-producing gastric cancer patients

**Survival data**	**AFPGCs, *n *(%)**	**Non-AFPGCs, *n *(%)**	**Studies, *n *(study number)**
Case number	12	111	1 (5)
Median survival time, months	18.7	41.3	
1-year survival rate, *N*	37	412	2 (2, 4)
	21 (58.0)	377 (91.4)	
3-year survival rate, *N*	37	412	2 (2, 4)
	4 (11.3)	248 (60.1)	
5-year survival rate, *N*	49	523	3 (2, 4, 5)
	5 (9.4)	164 (31.3)	

AFPGC, α-fetoprotein-producing gastric cancer.

We conducted this evaluation to explore the characteristics of AFPGC and to compare AFPGC with non-AFPGC. We found that patients with AFPGC had larger tumor size, weaker cell differentiation, worse histopathological type, deeper serosal infiltration, more lymph node and liver metastases, more advanced stages, shorter survival time and more VEGF-positive expression than patients with non-AFPGC. These observations are consistent with the results from other studies of AFPGC. Overall, we have confirmed that AFPGC is a subtype of GC that carries a poorer prognosis than non-AFPGC.

A previous report found a 2.5% prevalence of AFPGC in 104 AFPGC patients in China [8], which is less than our results. The discrepancy might be due to the different methods used for the measurement of serum AFP level or to regional or racial differences. From among the 11 studies we reviewed, some involved the measurement of AFP level in serum and others measured AFP through immunohistochemical evaluation. We cannot distinguish the difference because of the incomplete data provided in these publications. Overall, the objective prevalence requires more repeated studies with larger sample sizes.

We found that more than 40% of AFPGC cases developed in the antrum of the stomach, which suggests the importance of detecting AFP level in GC at the antrum. Particularly, liver metastasis is a very important prognostic factor during the control of AFP-positive gastric cancer because 56.7% of AFPGC patients in our evaluation had liver metastasis. Liver metastasis leads to poor liver function, resulting in intolerance of various treatment modalities and unsatisfying quality of life.

The poor prognosis is observed at the condition of high AFP level because the tumor exhibits a tendency to metastasize to the liver, even if the tumor is no deeper than the submucosa [9-12]. Recurrence of the tumor in the liver after surgery is a major cause of poor outcomes.

More than 90% of AFPGC cases exhibit high expression of VEGF, which suggests that VEGF inhibitors can be used for the treatment of AFPGC. The treatment efficacy using VEGF inhibitors may be better than that in other subtypes of GC.

It should be noted that increasing AFP level could also be observed in damaged liver cells from alcoholics, patients with chronic liver cirrhosis and carriers of the surface antigen of the hepatitis B virus. Thus, it is important to exclude these patients from studies to eliminate the selection bias [13].

The molecular or cellular mechanisms resulting in aggressive clinical behavior and poor prognosis of AFPGC are still unclear. However, AFPGC is associated with higher proliferative activity, weaker apoptosis and more plentiful neovascularization because AFPGC has poor cell differentiation and positive expression of VEGF [7,14]. According to previous reports, the abnormal expression of CD10, caudal-type homeodomain transcription factor CDX. and c-Met may also play critical roles in the occurrence, development and aggressiveness of AFPGC [15,16].

We attempted to analyze the statistical differences between different groups; however, this investigation is very heterogeneous because of some missing data, so it is difficult to perform a meta-analysis. However, the presented data give an impression that the prognosis of AFPGC is actually poorer than that of non-AFPGC, which can provide some useful information for clinical physicians. Although there has been no systematic review or meta-analysis to date, we believe that our evaluation can provide an overall review of AFPGC.

## Conclusions

AFPGC is a subtype of GC with a high risk of rapid metastasis to the liver. Its aggressive behavior, as well as its unique clinicopathological features, should be explored further at the cellular and molecular levels to develop effective therapies in multiple modes. In this review, we did not present the therapeutic modalities because of the lack of standard treatment procedures. Further studies with larger sample sizes are needed.

## Abbreviations

AFP: α-Fetoprotein; GC: Gastric cancer; AFPGC: α-fetoprotein-producing gastric cancer; VEGF: Vascular endothelial growth factor.

## Competing interests

The authors declare that they have no competing interests.

## Authors’ contributions

XDL performed the literature search. XDL, MJ and JW collected and summarized the data. XDL and BL wrote the manuscript and submitted it for publication. HBS helped with manuscript editing. CPW and JTJ provided the review concept and guided the writing. All authors read and approved the final manuscript.
